# Exploring disease-specific metabolite signatures in hereditary angioedema patients

**DOI:** 10.3389/fimmu.2024.1324671

**Published:** 2024-04-25

**Authors:** Adine Kanepa, Jingzhi Fan, Dmitrijs Rots, Annija Vaska, Laura Ansone, Monta Briviba, Janis Klovins, Natalja Kurjane, Kristaps Klavins

**Affiliations:** ^1^ Riga Stradiņš University, Riga, Latvia; ^2^ Institute of Biomaterials and Bioengineering, Faculty of Natural Sciences and Technology, Riga Technical University, Riga, Latvia; ^3^ Baltic Biomaterials Centre of Excellence, Headquarters at Riga Technical University, Riga, Latvia; ^4^ Children’s Clinical University Hospital, Riga, Latvia; ^5^ Latvian Biomedical Research and Study Centre, Riga, Latvia; ^6^ Pauls Stradiņš Clinical University Hospital, Riga, Latvia

**Keywords:** hereditary angioedema, C1 inhibitor (C1-INH), metabolome, biomarkers, diagnosis

## Abstract

**Introduction:**

Hereditary angioedema (HAE) is a rare, life-threatening autosomal dominant genetic disorder caused by a deficient and/or dysfunctional C1 esterase inhibitor (C1-INH) (type 1 and type 2) leading to recurrent episodes of edema. This study aims to explore HAE patients’ metabolomic profiles and identify novel potential diagnostic biomarkers for HAE. The study also examined distinguishing HAE from idiopathic angioedema (AE).

**Methods:**

Blood plasma samples from 10 HAE (types 1/2) patients, 15 patients with idiopathic AE, and 20 healthy controls were collected in Latvia and analyzed using LC-MS based targeted metabolomics workflow. T-test and fold change calculation were used to identify metabolites with significant differences between diseases and control groups. ROC analysis was performed to evaluate metabolite based classification model.

**Results:**

A total of 33 metabolites were detected and quantified. The results showed that isovalerylcarnitine, cystine, and hydroxyproline were the most significantly altered metabolites between the disease and control groups. Aspartic acid was identified as a significant metabolite that could differentiate between HAE and idiopathic AE. The mathematical combination of metabolites (hydroxyproline * cystine)/(creatinine * isovalerylcarnitine) was identified as the diagnosis signature for HAE. Furthermore, glycine/asparagine ratio could differentiate between HAE and idiopathic AE.

**Conclusion:**

Our study identified isovalerylcarnitine, cystine, and hydroxyproline as potential biomarkers for HAE diagnosis. Identifying new biomarkers may offer enhanced prospects for accurate, timely, and economical diagnosis of HAE, as well as tailored treatment selection for optimal patient care.

## 1 Introduction

Hereditary angioedema (HAE) is a rare, life-threatening autosomal dominant genetic disorder caused by a deficient or dysfunctional C1 esterase inhibitor (C1-INH) (type 1 and type 2), leading to the overproduction of bradykinin and the development of recurrent subcutaneous or submucosal edema (1, 2). Angioedema primarily affects the extremities, the face, the upper airway, and the gastrointestinal tract (2, 3). The disease is potentially life-threatening due to laryngeal edema and is unresponsive to antihistamines and glucocorticoids (3). The insufficient awareness of HAE, its sporadic symptom presentation, and the non-specific characteristics of the condition lead to underdiagnosis and delays in accurate identification. Consequently, this results in increased morbidity and a diminished quality of life owing to the postponed initiation of suitable treatment (4). Despite the identification of biomarkers across numerous pathologies, only a limited selection is presently applied in clinical settings for the diagnosis of HAE (5). Until now, based on recommendations, C4 level, C1-INH level, and activity are used to diagnose HAE 1/2 (4). Test results that point to HAE 1/2 should be repeated in a certified laboratory because time, temperature, and sample handling significantly impact C1-INH level and activity, leading to potentially incorrect values, inaccurate diagnoses, and thus inappropriate treatment (4, 6–8). Moreover, no specific biochemical method exists for the exact diagnosis of HAE-nC1-INH. It can be confirmed only by genetic sequencing of target genes, which is an expensive and time-consuming examination (7). Metabolomics has seen rapid growth in recent years, owing to its high sensitivity and ability to cover a wide range of metabolites using non- or minimally invasive methods, such as blood serum, plasma, and urine. Metabolomics has already been successfully used as a diagnostic tool in newborn screening programs to identify patients and initiate prompt therapy (9). Consequently, researchers have shifted their focus to identifying metabolites associated with various diseases, such as cancer, metabolic disorders (e.g., diabetes), hereditary conditions, neurodegenerative disorders, and cardiovascular diseases, with the ultimate goal of earlier disease detection, improved therapy management, and personalized medicine (10–12). Exploring the metabolite profile of HAE, which is currently not well-defined, could lead to an improved comprehension of the disease’s underlying mechanisms and facilitate the identification of novel metabolic biomarkers. These biomarkers can enable quicker, more precise, and economical diagnosis of HAE.

## 2 Methods

### 2.1 Study design

10 patients diagnosed with HAE-C1-INH, 15 patients with idiopathic angioedema, and 20 healthy controls were included in the study (**Table 1**). The p-value for key variables between of the patient cohort and control subjects, was greater than 0.05, indicating no statistically significant differences between groups. Diagnosis of HAE was based on personal and family history of angioedema and complement C4 levels, C1-INH levels, C1-INH functional activity, and genetic findings according to the global WAO/EAACI guideline definition 2021 (4). We asked for the following data for each patient: gender, date of birth, height, weight, the annual frequency of edema attacks, localization of angioedema, and the presence of other chronic diseases, including diabetes, autoimmune diseases, and cancer. Idiopathic angioedema was diagnosed by failure to determine the etiology with ≥ 3 attacks in a 6–12 month period. The study was approved by the Latvian Central Medical Ethics Committee (No. 01-29.1/2878, approved on 03/06/2020) and conformed to the principles of the Declaration of Helsinki. All data were anonymized before statistical analysis. All patients or their legal representatives have signed informed consent.

**Table 1:** Characterization of the patient cohort and control subjects.

	HAE	Idiopathic AE	HCs
n	10	15	20
Female	9	14	20
Male	1	1	0
Median age (y)	55 (35-62)	49 (34-56)	47 (33-67)
Median BMI	26 (26-32)	26 (24-29)	26 (18-36)
Skin edema	10	15	NA
Gactrointestinal edema	8	0	NA
Laryngeal edema	8	0	NA
Anunal attack frequency	7 (2-39)	9 (2-13)	NA

Data are presented at the median (IQR) or n.

### 2.2 Sample processing

Blood samples from all patients were obtained at least 8 days after an angioedema attack. Blood samples were obtained by puncture superficial vein of the forearm from patients with HAE (types 1/2) during symptom-free periods, collected into an ethylenediamine tetraacetic acid (EDTA) containing BD Vacutainer Blood Collection tube.

Plasma separation was performed by centrifuging peripheral blood sample tubes at 4000 rpm, +4 C, for 15 minutes. The obtained plasma was transferred to −80°C within 30 minutes and stored until metabolite analysis. For metabolite extraction 200 µL of serum sample was mixed with 800 µL of methanol,vortexed for 10 seconds, shaken for 20 min at 450 rpm, and afterwards centrifuged for 10 min at 10000 g. 100 µL of sample extract was transferred to an empty sample tube and dried down with a centrifugal vacuum evaporator. The resulting dry residues were reconstituted in 200 µL of methanol, to which 20 µL of the isotopically labeled internal standard mix was added. The prepared samples were transferred into HPLC vials for subsequent LC-MS analysis.

### 2.3 LC-MS analysis

In this study, a set of 55 metabolite including amino acids, amino acid-related metabolites, and acylcarnitines were measured. These metabolites were selected as they are routinely screened via mass spectrometry (MS/MS) in clinical laboratories during newborn screening (13). By measuring these established markers, changes detected in their concentrations in HAE patients’ blood could potentially offer a readily implementable diagnostic tool for this inherited disorder.

The LC-MS analysis was performed on a Dionex 3000 HPLC system (Thermo Scientific) coupled with an Orbitrap Q Exactive (Thermo Scientific) mass spectrometer. For chromatographic separation an ACQUITY UPLC BEH Amide, 1.7 µm, 2.1x100 mm analytical column (Waters) equipped with a VanGuard: BEH C18, 2.1x5 mm pre-column (Waters) was used. The column temperature was set to 40°C, the sample injection volume was 2 µL. As mobile phase A - 0.15% formic acid (v/v) in water was used and as mobile phase B - 0.15% formic acid (v/v) in 85% acetonitrile (v/v) with 10 mM ammonium formate was used. The gradient elution with a flow rate of 0.4 mL/min was performed resulting in the total analysis time of 17 min. The Orbitrap Q Exactive (Thermo Scientific) mass spectrometer was operated in a positive electrospray ionization mode. The following parameters were used for the ion source: spray voltage 3.5 kV, aux gas heater temperature 400°C, capillary temperature 350°C, aux gas flow rate 12, sheat gas flow rate 50. The MS detection was performed in a full MS scan mode, the scan range was set to m/z 50 to 400, mass resolution 35000, AGC target 1e6, maximum IT 50 ms. The Trace Finder 4.1 software (Thermo Scientific) was used for data processing. A seven-point linear calibration curve with internal standardization and 1/x weighing was constructed for the quantification of the metabolites.

### 2.4 Data analysis

The metabolomics data analysis was performed with MetaboAnalyst 5.0. For data preprocessing, obtained concentrations were log-transformed, scaled by mean-centering, and each variable was divided through its standard deviation. ROC analysis was performed to using “Biomarker Analysis” module. During ROC analysis ratios MetaboAnalyst was used to compute ratios of all possible metabolite pairs and then choose top ranked ratios (based on p values) to be included in the data for further biomarker analysis. GraphPad Prism 9 was used for statistical analysis. Analysis of variance (ANOVA) was applied for multi-group data, and fold-change and p-value were calculated for two-group comparisons to find significantly changed metabolites.

## 3 Results and discussion

**Figure 1:**
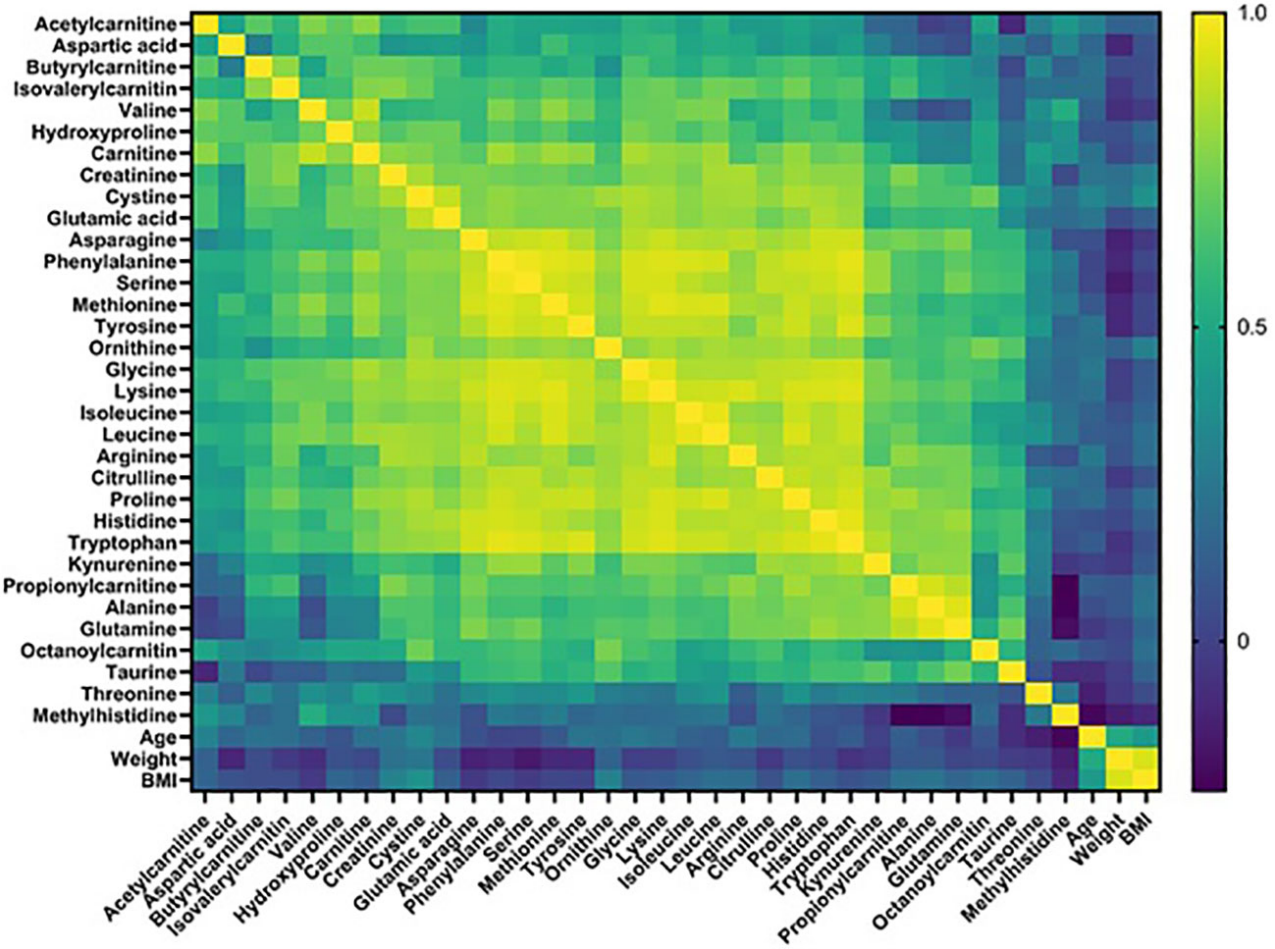
Correlation heatmap.

We conducted a quantitative targeted metabolomics study using liquid chromatography-mass spectrometry (LC-MS) using blood plasma samples from 10 HAE (types 1/2) patients, 15 patients with idiopathic AE, and 20 healthy controls (**Table 1**) collected in Latvia (14). Idiopathic AE was used as one of the control groups to exclude factors (e.g. NSAIDs, ACEIs, co-morbidities) that could alter the metabolome results. Out of 52 target metabolites, 33 were detected and quantified in all samples, including amino acids, acylcarnitines, and biogenic amines. Metabolomics data analysis was performed using MetaboAnalyst 5.0 and GraphPad Prism 9.0 software. Principal component analysis (PCA) score plots did not reveal distinct clustering patterns among different groups, indicating that the overall metabolite profiles of each group were similar. Furthermore, no significant correlations were found between the detected metabolite levels and the phenotypic data (**Figure 1**).

**Figure 2:**
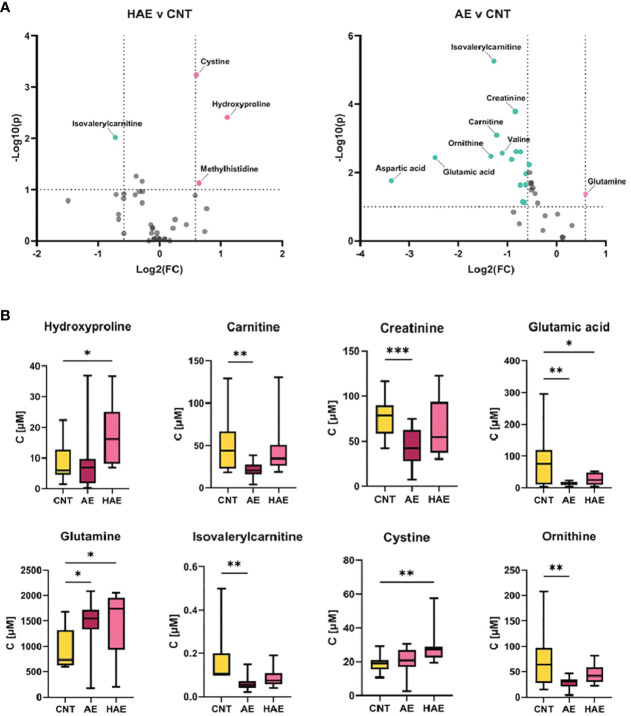
Significantly changed metabolites. **(A)** Volcano plots depicting fold change (FC) and p-value of metabolite concentration in different sample groups, thresholds: log2(fold change)>2 or <-2, and -log10(p)>1. **(B)** Bar plots for most significantly changed metabolites (*p-value < 0.05, **p-value <0.005, ***p-value <0.0005).

**Figure 3:**
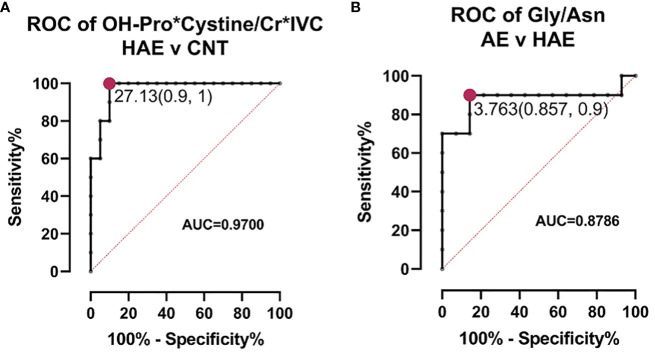
ROC analysis for biomarkers. **(A)** ROC curve of biomarker signature to distinguish between HAE and healthy controls (CNT). The cutoff value with highest sensitivity and selectivity indicated. **(B)** ROC curve of biomarker signature to distinguish between AE and HAE. The cutoff value with highest sensitivity and selectivity indicated.

We employed T-test and fold change calculations to identify differentially expressed metabolites between the two types of angioedema and the control group. The volcano plots in **Figure 2** highlight the significantly altered metabolites between the groups. Among these, isovalerylcarnitine, cystine, and hydroxyproline were identified as key metabolites that differed between both types of angioedema and the control group. Notably, cystine was particularly distinct in the HAE group, exhibiting a markedly lower level than the control group. Cystine, a sulfur-containing amino acid formed by the oxidation of two cysteine molecules, possesses antioxidant properties and plays a critical role in immune function (15). Decreased cystine levels have been found in cystinuria patients, a genetic disorder that impairs the reabsorption of specific amino acids in the kidneys (16). Patients from both angioedema groups exhibited lower levels of isovalerylcarnitine, which is involved in the transport of fatty acids into cells for energy production and reflects the metabolism of valine, isoleucine, and leucine (17). It is important to note that our study’s results for isovalerylcarnitine (Carnitine-C5) are consistent with a recent investigation on HAE urine samples (12). Wang et al. reported that carnitine-C5 was markedly altered in HAE patients compared to healthy controls (HCs), with average metabolite levels decreasing by 0.8-fold change among HAE patients. The concurrence of our findings with the previous study supports the potential of carnitine-C5 as a promising biomarker for HAE. It further emphasizes the importance of metabolomics in disease research and underscores the need for multicenter efforts for comprehensive metabolite profiling to identify potential biomarkers. Hydroxyproline and its two isomeric forms, trans-4-hydroxy-L-proline and trans–3-hydroxy-L-proline, are vital in collagen synthesis and thermodynamic stability. Elevated hydroxyproline levels have been reported in conditions such as graft versus host disease, keloids, and vitiligo (18). Altered hydroxyproline levels are also linked to hereditary diseases such as epidermolysis bullosa, which causes fragile skin (19). Additionally, hydroxyproline is a biomarker of bone resorption; increased urinary hydroxyproline levels indicate collagen degradation in bones and are associated with osteoporosis (20). Interestingly, aspartic acid levels were higher in HAE patients than in idiopathic AE patients, indicating that aspartic acid is a significant metabolite that can differentiate between HAE and idiopathic AE. Aspartic acid is involved in the urea cycle, a metabolic pathway that removes excess nitrogen from the body. To evaluate the predictive value of individual metabolites, we conducted a receiver operating characteristic (ROC) analysis (**Figure 3**). Of the 11 metabolites that exhibited an area under the curve (AUC) greater than 0.7, combining multiple metabolites significantly improved the AUC values, thereby increasing the sensitivity and selectivity of potential biomarker signatures. Specifically, the ratio of (OH-Pro x Cystine)/(Cr x IVC), with a cutoff of >27.13, can serve as a diagnostic criterion for HAE, with a sensitivity of 100% and a selectivity of 90%. Similarly, a Gly/Asn ratio with a cutoff of >3.763 can be used as a diagnostic criterion for distinguishing idiopathic AE and HAE, with a sensitivity of 90% and a selectivity of 85.7%. It is worth noting that metabolites with significantly altered levels between the groups are not affected by age (21).

The current study has certain limitations that should be taken into consideration. Although all patients from Latvia diagnosed with HAE (types 1/2) were included in the study, the number of patients is relatively small due to the rarity of HAE and low awareness among physicians. However, the identification of new biomarker signatures and the results of our study have the potential to enhance the diagnosis of HAE, enabling earlier and more effective recognition of the disease. To validate the significance of our findings, it is recommended that a larger study group is included, preferably by combining cohorts from different countries. It is desirable to compare the results with other causes of AE. Such an approach would provide an opportunity to examine the metabolic changes related to the type of HAE, the pathogenic variation, and the severity of the disease. Furthermore, this would enable the prediction of the onset, severity, and treatment response of HAE attacks, ultimately leading to better clinical management of the disease. We strongly encourage other researchers to consider including metabolomics in HAE research. The metabolite panel utilized in this study is a standard tool for new-born screening and is expected to be widely applicable in other laboratories, including clinical settings.

In summary, despite the limitations of our study, our results provide a valuable contribution to the HAE diagnosis and treatment field. The potential benefits of identifying new biomarkers and conducting further research with larger study groups are significant and could significantly improve the lives of patients affected by this rare disease.

## Data availability statement

The datasets presented in this study can be found in online repositories. The names of the repository/repositories and accession number(s) can be found below: MTBLS8132 (Metabolights).

## Ethics statement

The studies involving humans were approved by Latvian Central Medical Ethics Committee. The studies were conducted in accordance with the local legislation and institutional requirements. The participants provided their written informed consent to participate in this study.

## Author contributions

AK: Formal Analysis, Investigation, Methodology, Writing – original draft. JF: Formal Analysis, Software, Visualization, Writing – review & editing. DR: Conceptualization, Writing – review & editing. AV: Investigation, Methodology, Software, Writing – review & editing. LA: Methodology, Writing – review & editing. MB: Methodology, Writing – review & editing. JK: Methodology, Writing – review & editing. NK: Conceptualization, Funding acquisition, Project administration, Supervision, Writing – review & editing. KK: Conceptualization, Investigation, Methodology, Supervision, Writing – review & editing, Writing – original draft.
